# Remote Monitoring of Patients Undergoing Transcatheter Aortic Valve Replacement: A Framework for Postprocedural Telemonitoring

**DOI:** 10.2196/cardio.9075

**Published:** 2018-03-16

**Authors:** Mathilde C Hermans, Martijn S Van Mourik, Hermie J Hermens, Jan Baan Jr, Marije M Vis

**Affiliations:** ^1^ Heart Centre Academic Medical Centre University of Amsterdam Amsterdam Netherlands; ^2^ Department of Technical Medicine University of Twente Enschede Netherlands; ^3^ Biomedical Signals and Systems Group University of Twente Enschede Netherlands

**Keywords:** transcatheter aortic valve replacement, postoperative care, electrocardiography, telemonitoring, telemedicine

## Abstract

**Background:**

The postprocedural trajectory of patients undergoing transcatheter aortic valve replacement (TAVR) involves in-hospital monitoring of potential cardiac rhythm or conduction disorders and other complications. Recent advances in telemonitoring technologies create opportunities to monitor electrocardiogram (ECG) and vital signs remotely, facilitating redesign of follow-up trajectories.

**Objective:**

This study aimed to outline a potential set-up of telemonitoring after TAVR.

**Methods:**

A multidisciplinary team systematically framed the envisioned telemonitoring scenario according to the intentions, People, Activities, Context, Technology (iPACT) and Functionality, Interaction, Content, Services (FICS) methods and identified corresponding technical requirements.

**Results:**

In this scenario, a wearable sensor system is used to continuously transmit ECG and contextual data to a central monitoring unit, allowing remote follow-up of ECG abnormalities and physical deteriorations. Telemonitoring is suggested as an alternative or supplement to current in-hospital monitoring after TAVR, enabling early hospital dismissal in eligible patients and accessible follow-up prolongation. Together, this approach aims to improve rehabilitation, enhance patient comfort, optimize hospital capacity usage, and reduce overall costs. Required technical components include continuous data acquisition, real-time data transfer, privacy-ensured storage, automatic event detection, and user-friendly interfaces.

**Conclusions:**

The suggested telemonitoring set-up involves a new approach to patient follow-up that could bring durable solutions for the growing scarcities in health care and for improving health care quality. To further explore the potential and feasibility of post-TAVR telemonitoring, we recommend evaluation of the overall impact on patient outcomes and of the safety, social, ethical, legal, organizational, and financial factors.

## Introduction

### Transcatheter Aortic Valve Replacement and Cardiac Conduction Disorders

Transcatheter aortic valve replacement (TAVR) is a relatively new therapy for severe aortic valve stenosis, in which a valve prosthesis is positioned percutaneously within the diseased native aortic valve under radiological guidance [1].

Currently, the number of patients undergoing TAVR is growing fast, which stems from the increasing prevalence of aortic stenosis and the rising number of studies reporting similar to favorable outcomes for TAVR as compared to conventional valve surgery [2-4]. Furthermore, indications for TAVR are evolving to intermediate-risk patients and might even include low-risk patients in the future.

With this growth of TAVR procedures, optimization of patient outcome and the periprocedural trajectory is desired. Hence, prevention and adequate management of complications are key. Currently, one of the most common complications after TAVR is the development of cardiac conduction defects (CCD) or arrhythmias, of which left bundle branch blocks and first or third degree atrioventricular blocks have been most commonly reported [3,5-7].

In 60-83% of the patients with new-onset CCDs after TAVR, the CCDs develop during the procedure or within 24 hours after the procedure [8-10]. However, CCDs can develop up to days or even weeks after the TAVR procedure, during which the incidence of new-onset CCDs decreases in time [11]. Due to the development of CCDs, implantation of a permanent pacemaker (PPI) may be required. The risk of PPI depends on various factors including, but not limited to, sex, preexistence of right bundle branch blocks, prosthesis dimensions, and mitral valve calcification [8,12-14]. According to a meta-analysis including 41 studies that included >11,000 patients, the average risk of PPI after TAVR is about 17%, but rates between 2-51% have been reported [15]. With this, the occurrence of CCDs is a prominent and costly issue of TAVR.

### Postprocedural Monitoring

To follow up on pacemaker dependency or CCDs, continuous bedside or ambulatory electrocardiogram (ECG) telemetry is an essential part of in-hospital TAVR patient monitoring. According to the “ACCF/AATS/SCAI/STS Expert Consensus Document on Transcatheter Aortic Valve Replacement,” continuous monitoring of the ECG for the purposes of pacemaker-dependency identification is currently recommended for a minimum of 72 hours, depending on the valve type [16]. However, TAVR enables fast postprocedural recovery and mobilization of the patient, which is already possible from several hours after the procedure in the absence of adverse events.

Furthermore, all other aspects of in-hospital postprocedural care, such as neurologic evaluation and administration of postoperative medication, are only protocol for 24 hours after the procedure [16]. Accordingly, ECG monitoring as protocol is often the only indication for prolongation of hospital stay after 1-2 days when no complication presents. This is a major concern as it conflicts with the belief that hospital stays should be minimized to promote rapid recovery [17].

Early hospital dismissal enables early resumption of the daily life routine of patients, which is expected to improve patient comfort, and supports re-establishment of a stable condition [18,19]. Furthermore, reduction of length of stay can minimize the susceptibility for hospital-acquired complications and minimize the burden on hospital capacity. Driven by these advantages, fast-track protocols aiming for early ambulation are currently being developed for the TAVR population [20]. Yet, even with the introduction of fast-track routines, postprocedural ECG monitoring remains an obstacle for timely discharge. Therefore, new approaches of patient monitoring rejecting the need for hospital stays are warranted.

### Remote Patient Monitoring

In recent decades, global digitalization and the development of mobile medical technologies have increased the ability to transfer health-related data from one place to another and to perform physiological measurements in an outpatient setting. A particularly interesting application of mobile health is telemonitoring, in which mobile sensor applications facilitate remote follow-up of physiological parameters. Accordingly, telemonitoring systems that track vital parameters can create alternative strategies for current in-hospital monitoring. With this approach, patients are no longer confined to the hospital for follow-up of the ECG or other vital signs, which opens doors to redesigning the postprocedural patient trajectory.

For the TAVR population, the introduction of remote monitoring technologies raises the possibility of shortening hospital stay length in eligible patients without abstaining from follow-up of pacemaker dependency. As mentioned previously, this can promote fast rehabilitation, procure a patient-friendly postprocedural trajectory, and optimize use of hospital bed capacity.

Further, remote measurements of the ECG waveform and additional parameters including respiratory rate and activity level may help detect overall physical deteriorations in an outpatient setting. Thus, the monitoring intensity and period can be personalized easily. By adding this more continuous monitoring to the intermittent controls of conventional post-hospital follow-up, late-onset CCDs as well as physical deteriorations can be detected earlier and be acted on quickly, which could prevent further worsening and re-admissions. This approach can be valuable on its own in any patient and also complement the remote follow-up of patients dismissed early.

Together, it is expected that the introduction of telemonitoring can improve patient outcome and enhance patient comfort. Moreover, telemonitoring can increase the efficiency of patient care and reduce overall costs, which is critical to cope with the growing demand on health care. So, there is potential, but the question is how to use and select such new technology, for whom is it suited, and how telemonitoring should be embedded in clinical routine.

In this paper, we outline a potential scenario for the use of telemonitoring after TAVR, assess technological components that are needed, and evaluate factors that are essential for the implementation of this new approach to patient management.

## Methods

A multidisciplinary team was put together of medical and technical professionals with expertise on TAVR patient management, cardiac monitoring, CCDs, and telemonitoring. The expert team consisted of 2 interventional cardiologists, of which one was working as head of the TAVR team and the other as clinical chief of the cardiac monitoring unit; an electrophysiologist; and 2 technical physicians from the Academic Medical Center (Amsterdam, The Netherlands), which holds a center of expertise for TAVR. A professor in telemedicine from the University of Twente (Enschede, The Netherlands) also joined the team.

The expert team assembled to discuss and frame a potential future application of telemonitoring after TAVR using the early phase requirement elicitation methodology described by Larburu et al [21]. This scenario-based approach is specifically designed for telemedicine applications and is suitable for a multidisciplinary collaboration of medical practitioners and engineers as it promotes a mutual understanding of the desired user activity and user-system interactions. Accordingly, a concept scenario was defined using the “intentions, People, Activities, Context, and Technological components” (iPACT) framework for telemonitoring.

Next, the “Functionality, Interaction, Content, and Services” (FICS) descriptions were assessed, which were used to identify relevant user-system interactions and system functionalities. The iPACT and FICS were formulated using observations in current practice, international guidelines, and experiences and visions of the expert team members. Using the iPACTS and FICS as endpoints, the main requirements of the telemonitoring system were specified.

## Results

### Envisioned Scenario

**Figure 1:**
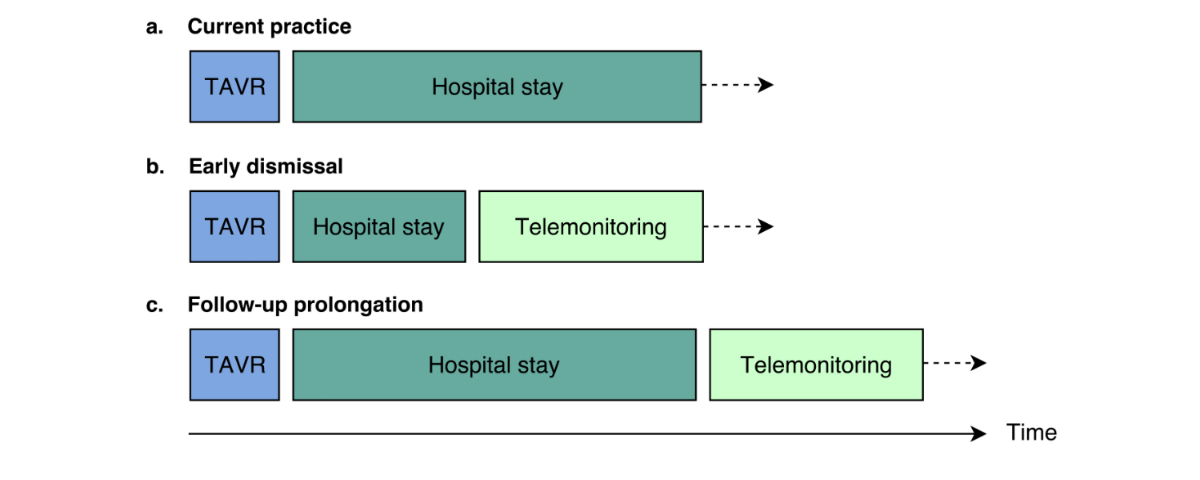
Presentation of the pathway following transcatheter aortic valve replacement (TAVR) in current practice; in the setting of early dismissal involving telemonitoring as partial replacement of current hospital stay; and in the setting of follow-up prolongation using telemonitoring subsequent to current hospital stay length.

The expert team agreed on an extensive scenario description framed by the iPACT and FICS and corresponding system criteria. The main principle of the formulated scenario described by the iPACT framework is that remote monitoring allows a reduction of hospital stay length by replacing in-hospital rhythm follow-up and facilitates prolongation of patient monitoring (Figure 1). The eventual goals (“Intentions”) of this approach are encouraging improved patient outcome and fast rehabilitation while increasing health care efficiency and cost reduction.

The suggested application of telemonitoring applies to patients who undergo TAVR and are subsequently admitted to the monitoring unit for postprocedural follow-up (“People”). Early hospital dismissal is indicated only in hemodynamically stable patients who have a low-risk profile for development of complications other than CCDs. 

Risk stratification may involve conventional methods such as the Society of Thoracic Surgeons predicted risk of mortality (STS-PROM) score or the European System for Cardiac Operative Risk Evaluation (EuroSCORE), or new risk scores specifically developed for the TAVR population and postprocedural setting [22,23]. Patients with a complicated procedure or admission may not be suitable for early dismissal. In eligible patients, hospital dismissal will be possible from the moment that rhythm observation is the only indication for prolongation of hospital stay.

Use of telemonitoring for additional monitoring after a conventional hospital stay can apply to any TAVR patient. However, follow-up prolongation is particularly recommended in patients with doubtful recovery or with increased risks for late development of CCDs or other complications.

**Figure 2:**
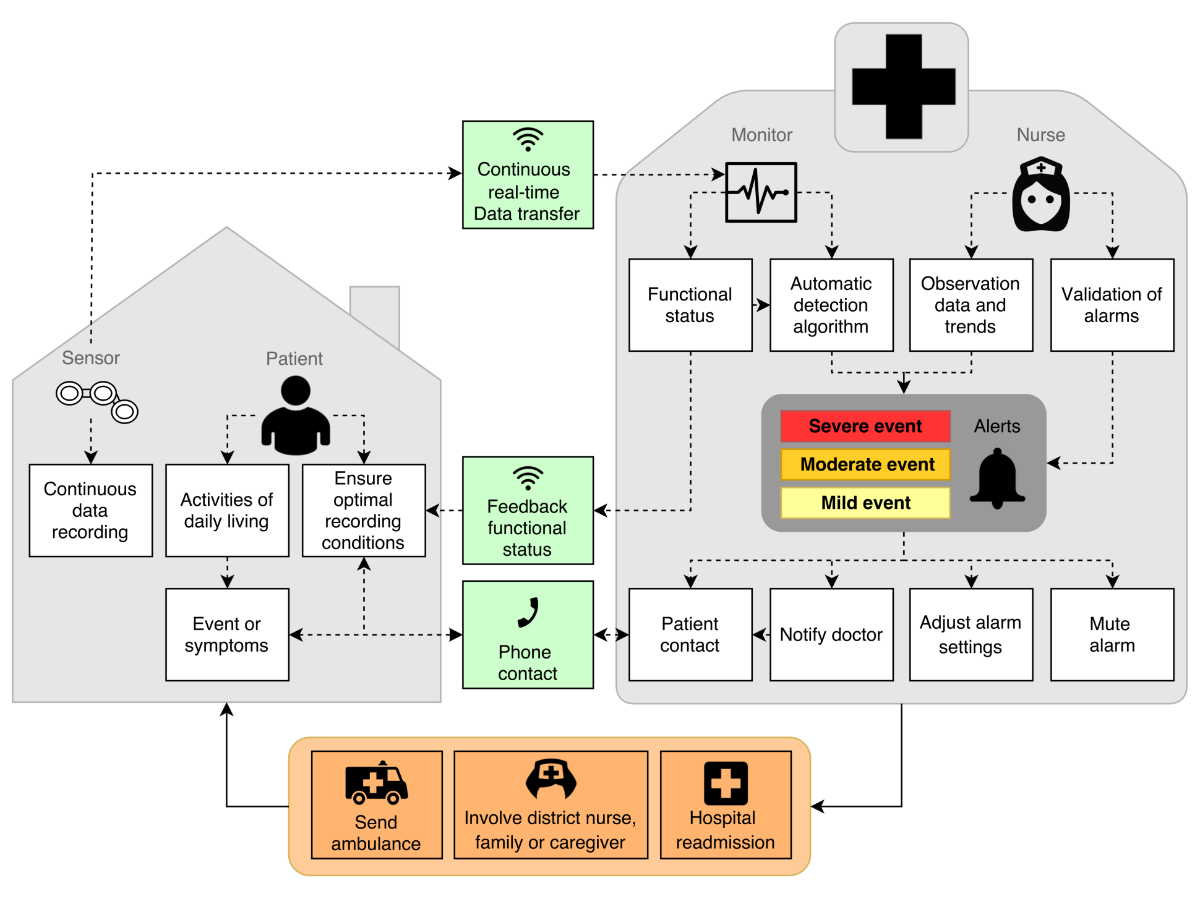
Overview of the suggested telemonitoring scenario.

The scenario describes the actions that are undertaken (“Activities”), in which setting (“Context”), and with which tools telemonitoring (“Technology”) is procured (Figure 2).

If patients are considered eligible for early dismissal or additional follow-up by the treating physician, they are equipped with a mobile sensor system that automatically provides continuous registration of the ECG in any ambulant setting. The system should also facilitate continuous registration and analysis of the respiratory rate and type or level of activity. Optionally, body temperature and blood pressure can be added, which are measured with connected arm cuff and thermometer. Last, incorporating a digital application with an interactive interface to allow symptom registration by the patient would be beneficial.

These measurements provide complementary information about the patient’s condition and recording setting, which may be valuable to contribute to ECG interpretation. Furthermore, these parameters may support trend analysis and trend identification of physical deteriorations leading to personalized (medical) advice. This is of particular interest for patients with congestive heart failure, kidney disease, or cardiorespiratory comorbidities where hemodynamic instability may occur. Furthermore, this approach might be desirable in patients where new valve types are implanted and where the risk of conduction disorders is still unknown.

In both applications of telemonitoring, the recorded ECG and additional data are continuously transferred to the central monitoring unit allowing real-time processing and patient monitoring conform clinical standards by dedicated care providers. To support the notification of events, the system automatically analyzes data and generates alerts in case of abnormalities and gradual deteriorations, adapted to the specific patient’s needs. Supported by this system, the patient can be dismissed from the hospital and resume the activities of daily life again while being monitored as long as needed. If the patient is not (yet) self-supportive, transfer to a convalescent home or additional home care can be considered.

With this envisioned approach, telemonitoring itself is essentially similar to standard telemetry monitoring despite the fact that the patient is no longer physically present within the hospital and that vital signs are assessed more continuously. However, the management of adverse events differs from current practice. For minor events or unforeseen system malfunctioning, the care provider may need to contact the patient by (video)phone to check the current physical status. Similarly, patients can contact or alert the monitoring unit when they are symptomatic by phone or by pushing an emergency button. The physician in charge can then decide to provide advice, recall the patient to hospital, or activate help from nearby caregivers if needed. In situations where the ECG shows increasing instability like prolongation of the atrioventricular conduction time interval or onset of atrial fibrillation [11], preventive measures can be taken such as further in-hospital observation and prescription of anticoagulants, respectively. This also applies to abnormal trends in vital signs, which may indicate development of cardiorespiratory instability. In case of emergencies, an ambulance team should be sent.

**Figure 3:**
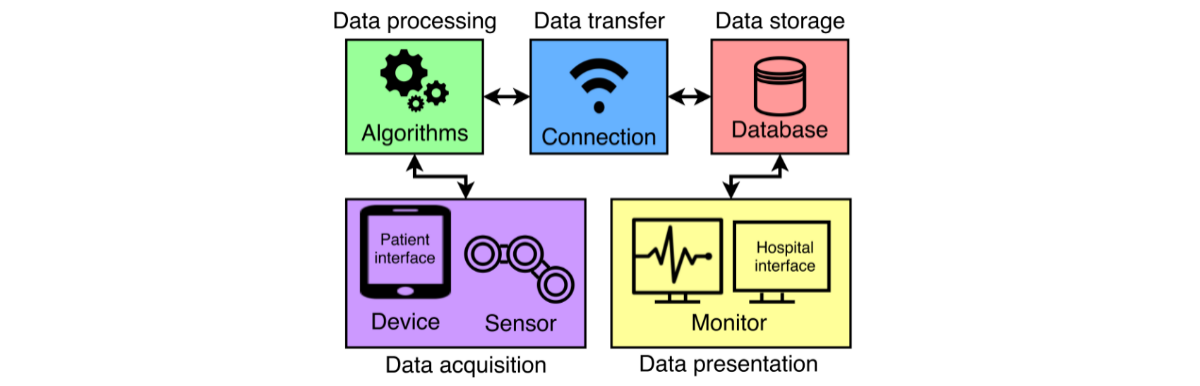
Overview of the processes and corresponding technical components of a telemonitoring system.

### Technology

**Figure 4:**
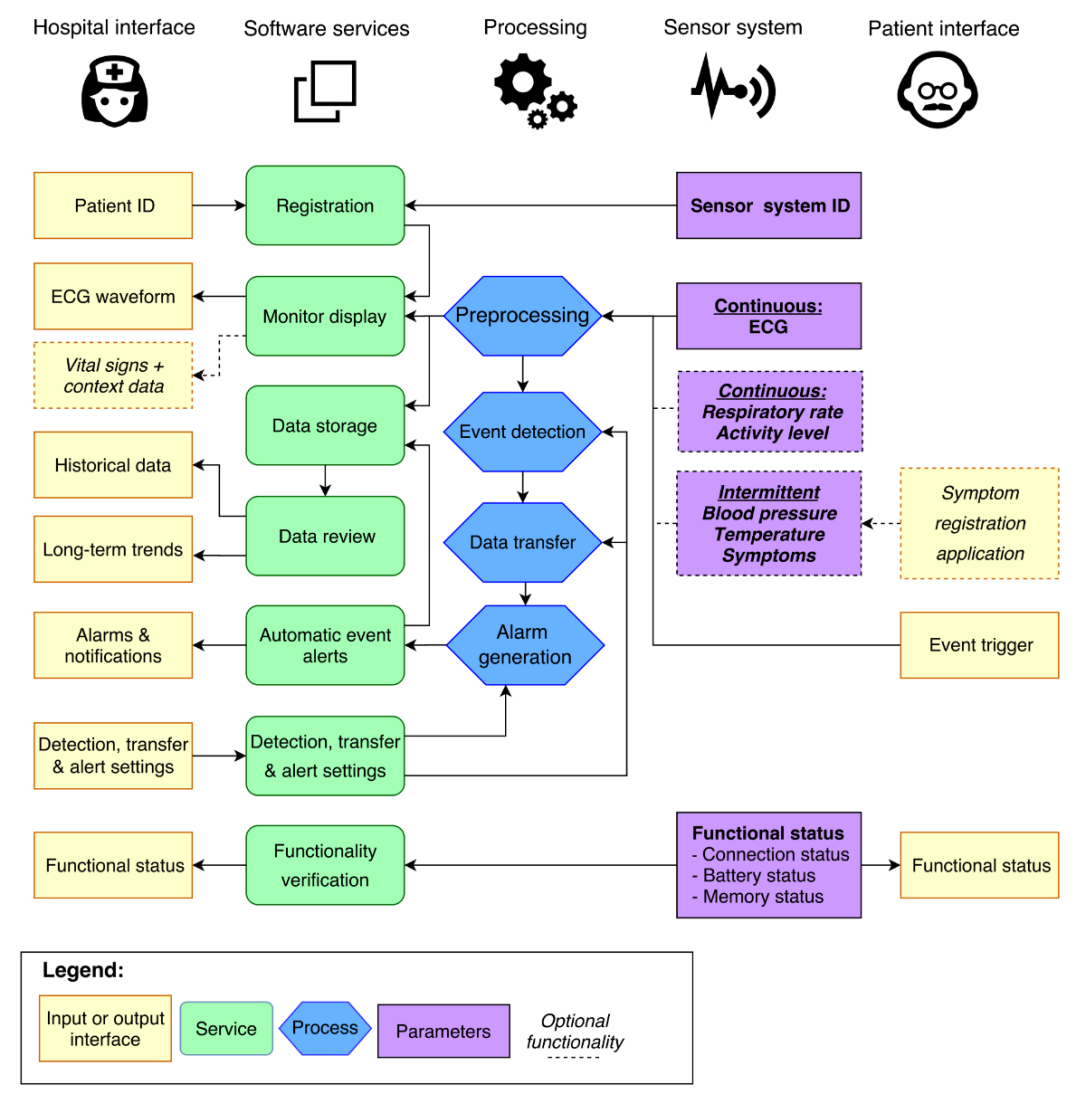
The processes facilitated by the telemonitoring system. ECG: electrocardiogram.

According to this scenario, the telemonitoring system should manage several processes and provide multiple services described by the FICS. In summary, these processes and services involve management of clinically, patient-, or system-related data (“Content”). Required processes (“Functions”) include data acquisition, data processing, data transfer, data storage, and data presentation. To procure these processes, the system should provide a sensor device, algorithms, wireless connection, database, and interfaces for patients and clinicians (Figure 3). All components should be embedded using software that manages the data processes (“Interactions”) to enable system control and patient observation (“Services”). The minimum required processes are shown in Figure 4.

### Technical Needs and Requirements

To provide a suitable alternative for in-hospital ECG monitoring, the telemonitoring system should primarily enable remote evaluation of pacemaker dependency. Second, the system should instantaneously notify development of cardiac arrhythmias and conduction defects that result in life-threatening or dangerous conditions to enable fast activation of acute care. According to these endpoints and desired system functionalities described by the iPACT and FICS, technological components should meet multiple criteria.

First, the system needs to provide clinically usable data. Accordingly, the mono- or multiple lead ECG should be of sufficient quality to identify rhythm and conduction disorders. Likewise, the additional parameters including respiratory rate and activity should be reliable and presented in such a way that interpretation of the ECG and patient’s status is supported. Next, the system should enable continuous real-time monitoring in a remote setting, in which a stable connection and robust data processing are essential. The sensor recordings should be transferred according to a protocol in which the continuous data are transferred directly in case of a detected event or in case the nurse or patient activates a trigger. Otherwise, the data are transferred periodically in batches every 10 minutes. These data provide the full ECG waveform and an average value of respiratory rate and activity level values calculated every 2 minutes. The reason for using periodic transfer instead of standard continuous transfer is limited power consumption. Additionally, this approach prevents an overload of information. With this, the observing nurse is less prone to information fatigue and can focus on critical issues more easily, which allows observation of multiple patients.

To support timely identification of rhythm or conduction defects, advanced validated algorithms must be incorporated that trigger alerts in case of ECG abnormalities at the patient site. Also warranted are additional algorithms to notify of the presence of deviant trends and abnormalities in vital signs. Although further research is required in order to specify alert conditions and effective detection methods, we suggest using analytical methods that assess not only the absolute values but also the time trends or patterns of vital signs to promote accuracy [24-26]. To improve abnormality detection, it might be useful to integrate information from different sources as well. For example, accelerometry may help anticipate motion artefacts, adapt the threshold for tachycardia or tachypnea during physical activity, or perform fall detection [27]. Last, the algorithms should be personalized, where threshold values are based on the patient’s history using static or self-learning methods. To prevent alarm fatigue and support fast response in urgent situation, alerts should be classified according to the priority level (ie, mild, moderate, severe events).

Since the patient has no support from health care professionals in their home environment, it is also important that the system is user friendly and comfortable to ensure correct use and acceptance by the TAVR population. Furthermore, the sensor system should be small and preferably worn under clothes, as visibility of the system could stigmatize the patient as being ill. Last, the system should be safe and ensure privacy. Specification of the requirements and corresponding priority level is provided for each of the technical components level in Multimedia Appendix 1. Together, these criteria require a combination of dedicated and advanced technology.

## Discussion

### Principal Consideration

In this approach to telemonitoring after TAVR, the ECG and patient’s status are evaluated remotely. Patients are no longer confined to the hospital for postprocedural rhythm observation, which facilitates early dismissal of eligible patients. Furthermore, telemonitoring enables prolongation of the monitoring period, promoting detection of late conduction disorders or physical deteriorations.

Telemonitoring is expected to positively impact patient outcome and patient comfort resulting in higher quality of care. In addition, it enables more optimal use of hospital bed capacity by relieving the burden on monitoring capacity. This approach suits the increasing (need for) decentralization of health care and can provide durable solutions to handle the increasing demand on health care with limited resources.

### Technological Potency

Ambulatory ECG monitoring itself is nothing new, as Holter and telemetry systems have been used for years. Yet, this suggested telemonitoring application requires more advanced mobile ECG systems in which continuous recordings, real-time data transfer, and remote use are integrated. With the fast expanding field of mobile health technologies and development of wireless networks, new solutions providing this feature combination seem to be entering the market [28,29]. Hence, it is likely that suitable telemonitoring systems will be available in the coming years. However, in our experience, currently available systems are not yet compliant with all requirements. This often relates to restricted battery duration of sensors, unsatisfactory data quality in remote settings, connection issues, or limited interoperability with current hospital systems. Also, many systems rely either on traditional rhythm detection methods or on threshold-based vital sign assessment, while the proposed application needs algorithms that take into account context and personal characteristics as well. In order to help telemonitoring systems find their place in health care settings, it is essential that all involved parties within and outside the hospital collaborate closely in system development and implementation.

### Feasibility

As depicted in this telemonitoring framework, successful implementation of remote patient monitoring does not only require suitable technology but involvement of the complete chain of health care delivery as well. With the relocation of health care, roles and responsibilities will be redistributed [30,31]. Although the concept of health care decentralization is gaining support worldwide, most health care systems are not yet ready for this approach [32,33]. As a result, various safety, organizational, financial, legal, and social issues may need to be addressed before telemonitoring can become embedded.

To facilitate telemonitoring, the organizational structure and workflow of first and second line health care providers need to be adapted. To start with, this requires allocation of a nurse for observation, organization of a process for routine distribution and technical maintenance of monitoring systems, and establishments of protocols for all involved caregivers. Correspondingly, the legal responsibilities have to be reevaluated in which the interposition of physical distance between patient and physician and the increased dependency on technology have to be taken into account.

In terms of social impact, there needs to be investigation into what extent the patient and their family support transfer of monitoring activities in a home environment, as this may require a certain level of self-support and anticipation of technology. This also applies to medical professionals, who need to be willing to adopt novel manners of patient management.

Last, to finance the implementation of telemonitoring, further clarification of the distribution of financial expenses and profits is needed to identify structural yields and find investors. In this process, the reimbursement policy plays a critical role, which varies per country [34].

### Patient Safety

Patient safety is a serious issue in remote patient practices. Post-TAVR telemonitoring can enhance patient safety when used for prolongation of the conventional monitoring period. However, safety may be a factor if the physical distance between patient and caregiver delays delivery of care. The actual overall risk may be limited as CCDs mostly present within the first 24 hours after TAVR and rarely lead to emergency situations where immediate action is required. In addition, current follow-up policy does not prevent certain events occurring after hospital dismissal either. Altogether it is a matter of creating minimal risks, which underlines the importance of easily accessible follow-up tools and careful selection of patients eligible for early discharge. Nevertheless, it is essential that telemonitoring be implemented step-by-step, with safety constantly evaluated. Furthermore, use of strict protocols and establishment of collaboration with professional emergency teams or remote first line caregivers is critical.

### Limitations

By assembling a multidisciplinary expert team representing various medical and technical disciplines, we pursued proposition of an adequate and realizable telemonitoring concept. Yet despite the promising prospects, it is not clear to what extent the intended goals will be obtained in real practice. Further research regarding the effectiveness of this concept is required, involving evaluation of the overall effects on patient outcome, efficiency, and cost-effectiveness. Furthermore, multicenter studies are recommended to assess the need for center-specific adaptation of the scenario or system requirements.

### Future Perspective

The introduction of telemonitoring provides the opportunity to relocate follow-up of ECG and vital signs, which supports reorganization of the postprocedural follow-up trajectory. Meanwhile, alternative approaches for post-TAVR monitoring are considered as well, such as the introduction of risk profiles to select patients for telemetry [11,35]. Although this approach is of potential interest to reduce redundant telemetry use, this may be applicable to only a part of the TAVR population. To verify the most optimal strategy for ECG follow-up, further evaluation of patient eligibility, patient safety, and efficiency is required.

To expand the value of telemonitoring, the remote system may incorporate extra services for patients such as training exercises or medication alerts to enhance rehabilitation and therapy loyalty. Additionally, creating a secured platform where patients can access and share their historical data can promote patient empowerment. The proposed telemonitoring set-up can be applied as an unobtrusive alternative for in-hospital telemetry or for real-time or off-line monitoring of many other patient groups. This may be valuable for postprocedural monitoring, therapeutic guidance, patient coaching, and many other diagnostic or therapeutic aims.

### Conclusions

Overall, the introduction of telemonitoring creates the opportunity to redesign the postprocedural trajectory of TAVR patients, enabling early hospital dismissal in eligible patients or prolongation of the period of monitoring in a daily life setting. To the end, this approach may not only be beneficial to improve health care quality but also inevitable to manage the increasing health-care demand and decentralization. Therefore, we recommend further exploration of the technical possibilities, optimal implementation, and overall impact at clinical, social, ethical, legal, safety, organizational, and financial levels. Accordingly, solutions may arise for more efficient and rehabilitation-supporting postprocedural monitoring that favor hospitals and patients.

## Appendix

Multimedia Appendix 1 System requirements.

## Abbreviations

CCD: cardiac conduction defects
ECG: electrocardiogram
FICS: Functionality, Interaction, Content, and Services
iPACT: intentions, People, Activities, Context, and Technology
PPI: permanent pacemaker implantation
TAVR: transcatheter aortic valve replacement

## References

[1] Leon MB, Smith CR, Mack M, Miller DC, Moses JW, Svensson LG, Tuzcu EM, Webb JG, Fontana GP, Makkar RR, Brown DL, Block PC, Guyton RA, Pichard AD, Bavaria JE, Herrmann HC, Douglas PS, Petersen JL, Akin JJ, Anderson WN, Wang D, Pocock S, PARTNER Trial Investigators (2010). Transcatheter aortic-valve implantation for aortic stenosis in patients who cannot undergo surgery. N Engl J Med.

[2] Osnabrugge RLJ, Mylotte D, Head SJ, Van Mieghem NM, Nkomo VT, LeReun CM, Bogers AJJC, Piazza N, Kappetein AP (2013). Aortic stenosis in the elderly: disease prevalence and number of candidates for transcatheter aortic valve replacement: a meta-analysis and modeling study. J Am Coll Cardiol.

[3] Mack MJ, Leon MB, Smith CR, Miller DC, Moses JW, Tuzcu EM, Webb JG, Douglas PS, Anderson WN, Blackstone EH, Kodali SK, Makkar RR, Fontana GP, Kapadia S, Bavaria J, Hahn RT, Thourani VH, Babaliaros V, Pichard A, Herrmann HC, Brown DL, Williams M, Akin J, Davidson MJ, Svensson LG, PARTNER 1 trial investigators (2015). 5-year outcomes of transcatheter aortic valve replacement or surgical aortic valve replacement for high surgical risk patients with aortic stenosis (PARTNER 1): a randomised controlled trial. Lancet.

[4] Arora S, Misenheimer JA, Jones W, Bahekar A, Caughey M, Ramm CJ, Caranasos TG, Yeung M, Vavalle JP (2016). Transcatheter versus surgical aortic valve replacement in intermediate risk patients: a meta-analysis. Cardiovasc Diagn Ther.

[5] Osnabrugge RLJ, Head SJ, Genders TSS, van Mieghem NM, De Jaegere PPT, van der Boon RMA, Kerkvliet JM, Kalesan B, Bogers AJJC, Kappetein AP, Hunink MGM (2012). Costs of transcatheter versus surgical aortic valve replacement in intermediate-risk patients. Ann Thorac Surg.

[6] Vahanian A, Alfieri O, Andreotti F, Antunes MJ, Barón-Esquivias Gonzalo, Baumgartner H, Borger MA, Carrel Tp, De Bonis M, Evangelista A, Falk V, Iung B, Lancellotti P, Pierard L, Price S, Schäfers H-J, Schuler G, Stepinska J, Swedberg K, Takkenberg J, Von Oppell UO, Windecker S, Zamorano Jl, Zembala M, Joint Task Force on the Management of Valvular Heart Disease of the European Society of Cardiology (ESC), European Association for Cardio-Thoracic Surgery (EACTS) (2012). Guidelines on the management of valvular heart disease (version 2012). Eur Heart J.

[7] Kodali SK, Williams MR, Smith CR, Svensson LG, Webb JG, Makkar RR, Fontana GP, Dewey TM, Thourani VH, Pichard AD, Fischbein M, Szeto WY, Lim S, Greason KL, Teirstein PS, Malaisrie SC, Douglas PS, Hahn RT, Whisenant B, Zajarias A, Wang D, Akin JJ, Anderson WN, Leon MB, PARTNER Trial Investigators (2012). Two-year outcomes after transcatheter or surgical aortic-valve replacement. N Engl J Med.

[8] Boerlage-Van DK, Kooiman KM, Yong ZY, Wiegerinck EMA, Damman P, Bouma BJ, Tijssen JGP, Piek JJ, Knops RE, Baan J (2014). Predictors and permanency of cardiac conduction disorders and necessity of pacing after transcatheter aortic valve implantation. Pacing Clin Electrophysiol.

[9] Guetta V, Goldenberg G, Segev A, Dvir D, Kornowski R, Finckelstein A, Hay I, Goldenberg I, Glikson M (2011). Predictors and course of high-degree atrioventricular block after transcatheter aortic valve implantation using the CoreValve Revalving System. Am J Cardiol.

[10] Bagur R, Rodés-Cabau J, Gurvitch R, Dumont É, Velianou JL, Manazzoni J, Toggweiler S, Cheung A, Ye J, Natarajan MK, Bainey KR, DeLarochellière R, Doyle D, Pibarot P, Voisine P, Côté M, Philippon F, Webb JG (2012). Need for permanent pacemaker as a complication of transcatheter aortic valve implantation and surgical aortic valve replacement in elderly patients with severe aortic stenosis and similar baseline electrocardiographic findings. JACC Cardiovasc Interv.

[11] Toggweiler S, Stortecky S, Holy E, Zuk K, Cuculi F, Nietlispach F, Sabti Z, Suciu R, Maier W, Jamshidi P, Maisano F, Windecker S, Kobza R, Wenaweser P, Lüscher TF, Binder RK (2016). The Electrocardiogram After Transcatheter Aortic Valve Replacement Determines the Risk for Post-Procedural High-Degree AV Block and the Need for Telemetry Monitoring. JACC Cardiovasc Interv.

[12] Smith CR, Leon MB, Mack MJ, Miller DC, Moses JW, Svensson LG, Tuzcu EM, Webb JG, Fontana GP, Makkar RR, Williams M, Dewey T, Kapadia S, Babaliaros V, Thourani VH, Corso P, Pichard AD, Bavaria JE, Herrmann HC, Akin JJ, Anderson WN, Wang D, Pocock SJ, PARTNER Trial Investigators (2011). Transcatheter versus surgical aortic-valve replacement in high-risk patients. N Engl J Med.

[13] Roten L, Wenaweser P, Delacrétaz E, Hellige G, Stortecky S, Tanner H, Pilgrim T, Kadner A, Eberle B, Zwahlen M, Carrel T, Meier B, Windecker S (2010). Incidence and predictors of atrioventricular conduction impairment after transcatheter aortic valve implantation. Am J Cardiol.

[14] Nazif TM, Dizon JM, Hahn RT, Xu K, Babaliaros V, Douglas PS, El-Chami MF, Herrmann HC, Mack M, Makkar RR, Miller DC, Pichard A, Tuzcu EM, Szeto WY, Webb JG, Moses JW, Smith CR, Williams MR, Leon MB, Kodali SK, PARTNER Publications Office (2015). Predictors and clinical outcomes of permanent pacemaker implantation after transcatheter aortic valve replacement: the PARTNER (Placement of AoRtic TraNscathetER Valves) trial and registry. JACC Cardiovasc Interv.

[15] Siontis GCM, Jüni P, Pilgrim T, Stortecky S, Büllesfeld L, Meier B, Wenaweser P, Windecker S (2014). Predictors of permanent pacemaker implantation in patients with severe aortic stenosis undergoing TAVR: a meta-analysis. J Am Coll Cardiol.

[16] Holmes DR, Mack MJ, Kaul S, Agnihotri A, Alexander KP, Bailey SR, Calhoon JH, Carabello BA, Desai MY, Edwards FH, Francis GS, Gardner TJ, Kappetein AP, Linderbaum JA, Mukherjee C, Mukherjee D, Otto CM, Ruiz CE, Sacco RL, Smith D, Thomas JD (2012). 2012 ACCF/AATS/SCAI/STS expert consensus document on transcatheter aortic valve replacement. J Am Coll Cardiol.

[17] Kehlet H, Wilmore DW (2008). Evidence-based surgical care and the evolution of fast-track surgery. Ann Surg.

[18] Shepperd S, McClaran J, Phillips CO, Lannin NA, Clemson LM, McCluskey A, Cameron ID, Barras SL (2010). Discharge planning from hospital to home. Cochrane Database Syst Rev.

[19] Shepperd S, Doll H, Broad J, Gladman J, Iliffe S, Langhorne P, Richards S, Martin F, Harris R (2009). Early discharge hospital at home. Cochrane Database Syst Rev.

[20] Marcantuono R, Gutsche J, Burke-Julien M, Anwaruddin S, Augoustides J, Jones D, Mangino-Blanchard L, Hoke N, Houseman S, Li R, Patel P, Stetson R, Walsh E, Szeto W, Herrmann H (2015). Rationale, development, implementation, and initial results of a fast track protocol for transfemoral transcatheter aortic valve replacement (TAVR). Catheter Cardiovasc Interv.

[21] Larburu NR, Widya IA, Bults RGA, Hermens HJ, Napolitano C (2013). Early phase telemedicine requirements elicitation in collaboration with medical practitioners.

[22] O'Brien SM, Shahian DM, Filardo G, Ferraris VA, Haan CK, Rich JB, Normand SL, DeLong ER, Shewan CM, Dokholyan RS, Peterson ED, Edwards FH, Anderson RP, Society of Thoracic Surgeons Quality Measurement Task Force (2009). The Society of Thoracic Surgeons 2008 cardiac surgery risk models: part 2--isolated valve surgery. Ann Thorac Surg.

[23] Nashef SAM, Roques F, Michel P, Gauducheau E, Lemeshow S, Salamon R (1999). European system for cardiac operative risk evaluation (EuroSCORE). Eur J Cardiothorac Surg.

[24] Imhoff M, Kuhls S (2006). Alarm algorithms in critical care monitoring. Anesth Analg.

[25] Churpek MM, Adhikari R, Edelson DP (2016). The value of vital sign trends for detecting clinical deterioration on the wards. Resuscitation.

[26] Borowski M, Görges M, Fried R, Such O, Wrede C, Imhoff M (2011). Medical device alarms. Biomed Tech (Berl).

[27] Yoon SW, Min SD, Yun YH, Lee S, Lee M (2008). Adaptive motion artifacts reduction using 3-axis accelerometer in e-textile ECG measurement system. J Med Syst.

[28] Martínez-Pérez B, de la Torre-Díez I, López-Coronado M, Herreros-González J (2013). Mobile apps in cardiology: review. JMIR Mhealth Uhealth.

[29] Majumder S, Mondal T, Deen MJ (2017). Wearable Sensors for Remote Health Monitoring. Sensors (Basel).

[30] Miller EA (2003). The technical and interpersonal aspects of telemedicine: effects on doctor-patient communication. J Telemed Telecare.

[31] Fairbrother P, Pinnock H, Hanley J, McCloughan L, Sheikh A, Pagliari C, McKinstry B, TELESCOT programme team (2012). Continuity, but at what cost? The impact of telemonitoring COPD on continuities of care: a qualitative study. Prim Care Respir J.

[32] Dinesen B, Nonnecke B, Lindeman D, Toft E, Kidholm K, Jethwani K, Young HM, Spindler H, Oestergaard CU, Southard JA, Gutierrez M, Anderson N, Albert NM, Han JJ, Nesbitt T (2016). Personalized Telehealth in the Future: A Global Research Agenda. J Med Internet Res.

[33] Brunetti ND, Scalvini S, Molinari G (2016). Innovations in telemedicine for cardiovascular care. Expert Rev Cardiovasc Ther.

[34] Block PC (2015). Evolving TAVR, and the need for intelligent design. Catheter Cardiovasc Interv.

[35] Auffret V, Puri R, Urena M, Chamandi C, Rodriguez-Gabella T, Philippon F, Rodés-Cabau J (2017). Conduction Disturbances After Transcatheter Aortic Valve Replacement: Current Status and Future Perspectives. Circulation.

