# Supplementary material for: Remote Monitoring of Patients Undergoing Transcatheter Aortic Valve Replacement: A Framework for Postprocedural Telemonitoring
Source: JMIR Cardio. 2018 Mar 16;2(1):e9. doi: 10.2196/cardio.9075 (PMC6834331; doi:10.2196/cardio.9075)
Supplement: Multimedia Appendix 1 [file cardio_v2i1e9_app1.pdf]

SYSTEM REQUIREMENTS

Supplementary Table 1. General requirements of Telemonitoring system

Priority level	Requirement
**Required**	1. The telemonitoring system enables mobile home monitoring of the electrocardiogram<br>2. The telemonitoring system is certified as medical device for use in the intended medical application<br>3. The telemonitoring system meets national safety criteria<br>4. The telemonitoring system is validated for its functionality in the intended medical application<br>5. The telemonitoring system does not conflict the patients privacy
**Desired**	6. The telemonitoring system is functionally similar to/better than monolead telemetry<br>7. The daily costs of monitoring using the telemonitoring system are lower than the daily costs of hospital admittance<br>8. The telemonitoring system is robust<br>9. The telemonitoring system is easy to use for the elderly patient<br>10. The telemonitoring system is easy to use for the nurse and doctor<br>11. The telemonitoring system enables simultaneous monitoring of multiple patients<br>12. The telemonitoring system can provide feedback regarding its functionality<br>13. The telemonitoring system enables mobile home monitoring of other parameters for detection of other clinically relevant events

Supplementary Table 2. Requirements of individual components of telemonitoring system

System component	Priority level	Requirement
**Sensor system**	Required	1. The ECG sensor system measures continuous single- or multiple leads ECG<br>1.1. The sensor provides sufficient signal quality during daily life activities<br>1.2. The ECG sensor system provides at least a bipolar configuration<br>1.3. The ECG signal range is at least +- 5 mV.<br>1.4. The sample frequency is at least 100 Hz<br>1.5. The operational temperature is 15-40°C<br>1.6. The sensor is accurate (sensitivity of max ±500 μV)<br>1.7. The sensor is suitable for adult man and women of different posture and age<br>2. The ECG sensor system is suitable for mobile monitoring<br>2.1. The sensor system is light weight (<0.2 kg)<br>2.2. The sensor system is portable and worn on the body surface or clothes<br>2.3. Battery capacity or power supply allows an uninterrupted recording of at least 24 hours<br>3. The ECG sensor system is safe<br>3.1. The sensor system is certified as a medical device, according to national guidelines<br>3.2. The sensor system is compatible with internal pacemakers<br>3.3. The sensor system is compatible with defibrillators<br>3.4. The sensor system does not leads to physical injury<br>3.5. The sensor system does not affect the functionality of medical equipment surrounding the hospital bed<br>4. The ECG sensor system is hygienic<br>4.1. The sensor system can be cleaned or is disposable
	Desired	5. The ECG sensor system is easy to use for the patient<br>5.1. Attaching the sensor is easy<br>5.2. Removing the sensor is easy<br>6. The ECG sensor system is comfortable<br>6.1. The sensor system is non-invasive<br>6.2. The sensor system brings minimal pain, itchiness or stress

		6.3. The sensor can be worn under clothes<br>6.4. The sensor system does minimally limit the patients movement<br>7. The ECG sensor system is robust and durable<br>7.1. The sensor system is water or splash proof<br>7.2. The sensor system functionality is maintained during daily life activities<br>8. The ECG sensor system indicates the quality of recordings<br>9. The ECG sensor is hypo allergic<br>10. The sensor system enables recording of multiple lead ECG<br>11. The ECG sensor system has an event button for patients<br>12. The sensor system registers respiratory rate<br>13. The sensor system registers activity level and type<br>14. The sensor system registers body temperature and blood pressure, which may be measured intermittently using additional devices.<br>15. The sensor system provides a digital application for the patient for symptom registration
	Required	16. ECG abnormalities[1] automatically detected with at least 95% sensitivity and 75% specificity<br>17. Alarm thresholds are adjustable
	Desired	18. Artifacts are automatically detected<br>19. The signal is filtered and preprocessed
**Data transfer**	Required	20. Data is transferred with a wireless connection from any remote locations the hospital region<br>21. Data can be transferred continuously (maximum transfer intervals of 30 s) [2]<br>22. The maximum delay of data transfer (in case of emergencies) is 10 s<br>23. The data is buffered or saved for at least 4 h in case data transfer fails<br>24. Data transfers automatically<br>25. Data is encrypted according to national privacy regulations
	Desired	26. Direct data transfer can be activated remotely in the Hospital interface or by the patient<br>27. Data transfer intervals can be adjusted (minimal interval range between 30 s – 10 min)
**Data presentation**	Required	28. The Hospital Interface enables access to real-time ECG recordings from the observation monitor<br>29. The Hospital Interface enables retrospective access to the ECG waveforms, detected events and trends in heart rate and additional vital signs
	Desired	30. The Hospital Interface enables adaptation of alarm settings or thresholds<br>31. The Hospital Interface enables adaptation of settings of data presentation on the observation monitor<br>32. The Hospital Interface is easy to use for the nurse and doctor<br>33. The Hospital Interface shows basic functionality of the Telemonitoring system (battery status, connection status, data quality)<br>34. The Patient Interface shows basic functionality of the Telemonitoring system (connection status)<br>35. The Hospital Interface enables simultaneous presentation of multiple patients.
**Data storage**	Required	36. The recorded data, trends and events are securely saved and can only be accessed by authorized people<br>37. The data can be accessed by the medical center of treatment anytime, without mediation of another institutes or companies
	Desired	38. Events can be saved or printed for storage in the clinical record of the patient

ECG: Electrocardiogram. 1: ECG abnormalities include asystole, ventricular fibrillation, ventricular tachycardia, tachycardia, bradycardia (adjustable threshold), abrupt or gradual change in heart rate, ventricular pause, irregular heart rate, missed beats, and premature complexes. 2: An acceptable alternative for continuous monitoring includes decentralized assessment with direct and continuous data transfer in case of ECG abnormalities or in case of patient or nurs
